# Sequential conditioning-stimulation reveals distinct gene- and stimulus-specific effects of Type I and II IFN on human macrophage functions

**DOI:** 10.1038/s41598-019-40503-y

**Published:** 2019-03-27

**Authors:** Quen Cheng, Faraz Behzadi, Supriya Sen, Sho Ohta, Roberto Spreafico, Rosane Teles, Robert L. Modlin, Alexander Hoffmann

**Affiliations:** 1 Department of Microbiology, Immunology, and Molecular Genetics, University of California, Los Angeles, CA 90095 United States; 2 Department of Medicine, Division of Infectious Diseases, David Geffen School of Medicine, University of California, Los Angeles, CA 90095 United States; 3 Institute for Quantitative and Computational Biosciences, University of California, Los Angeles, CA 90095 United States; 4 Division of Dermatology, David Geffen School of Medicine, University of California, Los Angeles, CA 90095 United States

## Abstract

Macrophages orchestrate immune responses by sensing and responding to pathogen-associated molecules. These responses are modulated by prior conditioning with cytokines such as interferons (IFNs). Type I and II IFN have opposing functions in many biological scenarios, yet macrophages directly stimulated with Type I or II IFN activate highly overlapping gene expression programs. We hypothesized that a sequential conditioning-stimulation approach would reveal with greater specificity the differential effects of Type I and II IFN on human macrophages. By first conditioning with IFN then stimulating with toll-like receptor ligands and cytokines, followed by genome-wide RNA-seq analysis, we identified 713 genes whose expression was unaffected by IFN alone but showed potentiated or diminished responses to a stimulus after conditioning. For example, responses to the cytokine TNF were restricted by Type II IFN conditioning but potentiated by Type I IFN conditioning. We observed that the effects of IFN were not uniformly pro- or anti-inflammatory, but highly gene-specific and stimulus-specific. By assessing expression levels of key signal transducers and characterizing chromatin accessibility by ATAC-seq, we identify the likely molecular mechanisms underlying Type I and Type II-specific effects, distinguishing between modulation of cytoplasmic signaling networks and the nuclear epigenome that synergistically regulate macrophage immune responses.

## Introduction

Macrophages play multiple crucial roles in initiating and coordinating healthy immune responses, and their dysregulation is associated with pathologic processes ranging from atherosclerosis to the cytokine storm seen in sepsis. One of the key functions of macrophages is to sense signals from the environment, such as pathogen associated molecular patterns (PAMPs) and cytokines, and translate these environmental inputs into a coordinated response involving the expression of hundreds of genes^1,2^. The specific nature of this response depends not only on the type of signal but also on the tissue microenvironment and prior cytokine exposures. Stimulus-responsive gene expression programs in macrophages are thus context-dependent. The same environmental signal that elicits an inflammatory response in one context might be immunologically silent in another.

One of the best-defined examples of this context-dependence is the “M1/M2” paradigm of macrophage polarization^1,2^. Macrophages conditioned with interferon (IFN)-*γ* and lipopolysaccharide (LPS) have been termed “classically activated” M1 macrophages and are skewed towards a pro-inflammatory phenotype that favors killing of intracellular pathogens. In contrast, macrophages conditioned with cytokines such as interleukin (IL)-4 have been termed “alternative” M2 macrophages whose functions are predominantly immunomodulatory and are important for tissue repair. First described in the late 1990s, these M1/M2 polarization states are now viewed as extremes of a wide spectrum of macrophage phenotypes that are defined by their exposure to diverse cytokine microenvironments^3,4^. In this model, cytokines “condition” macrophages, and the conditioning regimen can either “prime” or “tolerize” cells, respectively potentiating or diminishing their response to a subsequent stimulus.

Alterations in the epigenome are the primary mechanism of this phenomenon^5^. For instance, exposure to either IFN*γ* or IL-4 leads to a gain of enhancers and increases in chromatin accessibility as measured by ChIP- and FAIRE-seq.^6,7^. Furthermore, prior IL-4 exposure inhibits the gain of IFN*γ*-mediated enhancers, illustrating that cross-repressive mechanisms exist amongst the various cytokines to which macrophages are exposed^8^. In addition to epigenetic changes, cytokine conditioning can affect signaling and transcription factor activity as an additional mechanism of priming or tolerance^8–10^. Altogether, there has been a paradigm shift towards understanding macrophage biology within this framework of conditioning and subsequent response to stimulation.

The IFNs have long been appreciated as fundamentally important cytokines in the mammalian immune system whose functions go beyond antiviral host defense^11^. IFN*γ*, as described above, has a well-appreciated role for activating macrophages and is required for immunity to intracellular pathogens such as tuberculosis and listeria^12,13^. Similarly, the Type I IFNs also play a substantial role in regulating myeloid cell function^14,15^. One of their roles in macrophages is thought to be the induction of an anti-inflammatory state that is in contrast to the pro-inflammatory role of Type II IFN^16,17^. However, others have also shown that Type I IFNs can promote inflammation, induce apoptosis, enhance antigen-presentation, and participate in signaling cross-talk with other cytokines like tumor necrosis factor (TNF)^18–21^.

In some human disease states, Type I and II IFNs do indeed have contrasting effects. In *Mycobacterium leprae* infection, patients with lepromatous type, a progressive form of leprosy, possess an IFN*β* signature in their skin lesions, while patients with the self-limiting tuberculoid form of leprosy have an IFN*γ* signature at the site of infection^22^. Similarly, IFN*β* inhibits while IFN*γ* enhances the control of *Mycobacterium tuberculosis* infection^23^. A variety of mechanisms have been proposed for the opposing roles of Type I and II IFN, such as IFN*β* leading to down-regulation of IL-12 and antimicrobial peptides through IL-10, or IFN*β* suppression of IFN*γ* receptor expression^22,24,25^.

Despite these contrasting physiological effects of Type I and II IFN *in vivo*, gene expression studies have found that Type I and Type II IFN have highly overlapping effects on the macrophage transcriptome^26,27^. These results appear insufficient to explain the biological differences, and they challenge the dichotomy that IFN*γ* is pro-inflammatory while IFN*β* is anti-inflammatory. Notably, however, these studies have assessed only the direct gene-expression consequences of IFN and have not addressed the physiologically relevant paradigm of macrophage conditioning followed by stimulation. Additionally, the majority of studies on macrophage conditioning have been done using murine macrophages, and data are lacking from human cells which are likely to be different^28,29^.

We therefore sought to define with high resolution the effects of Type I and II IFN on human macrophages using sequential conditioning and stimulation. We hypothesized that additional differences would be revealed by unbiased, genome-wide transcriptomic analyses of macrophages conditioned with IFN*β* or IFN*γ* and subsequently stimulated with various PAMPs and cytokines. Our findings reveal complex and nuanced differences between Type I and II IFNs that are gene-specific and stimulus-specific.

## Results

### Gene expression programs in human macrophages are stimulus-specific

**Figure 1:**
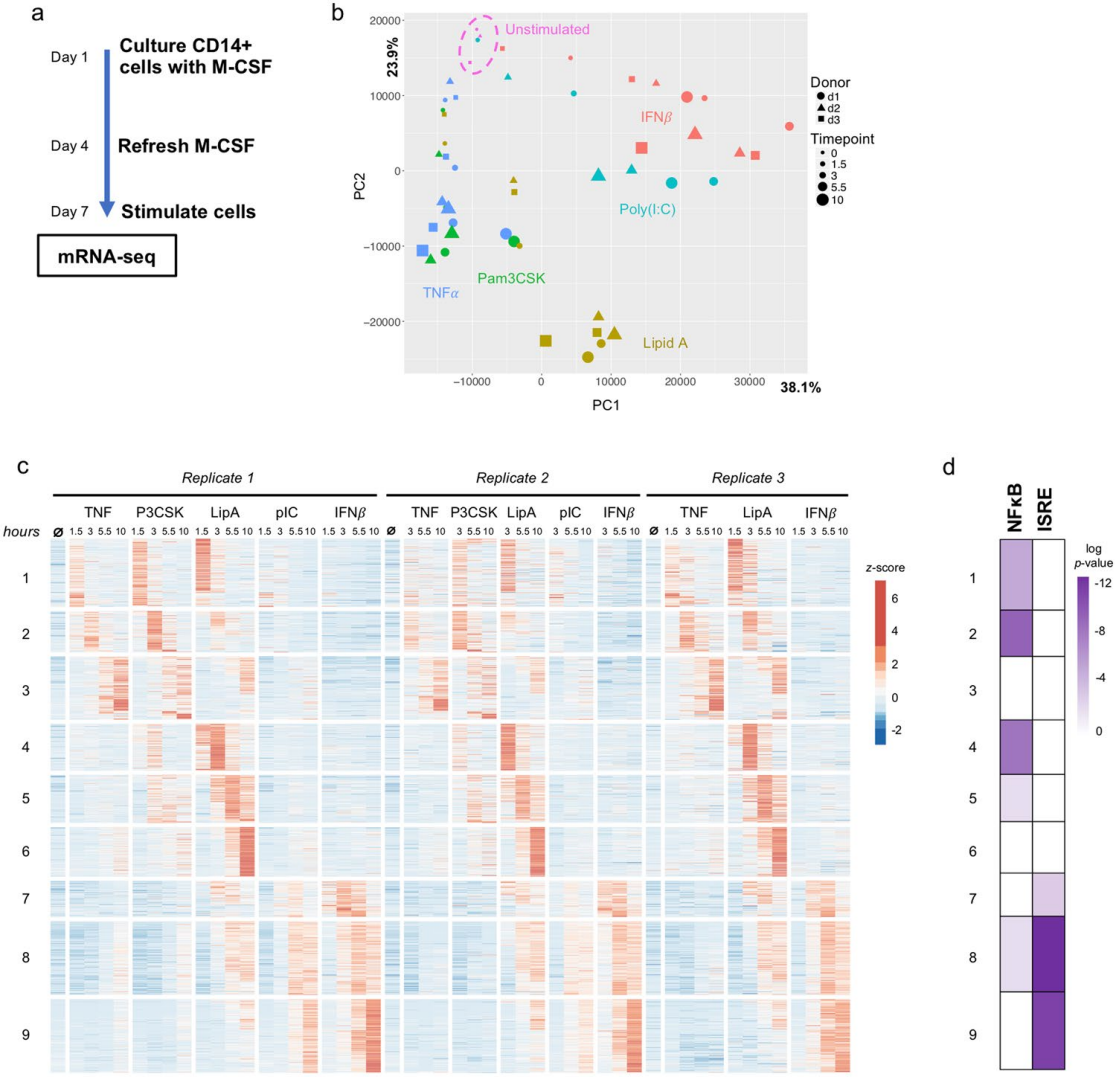
Stimulus-responsive gene expression in human macrophages. (**a**) Experimental design. (**b**) Principal component analysis of expressed genes in naïve macrophages with five stimulation conditions. (**c**) Heat map of 1421 genes induced at least four-fold by any stimulus (FDR < 0.05). (**d**) Transcription factor motif analysis for enrichment of NFκB and ISRE sequences within promoters of clustered genes.

To characterize the gene expression response of primary human macrophages we isolated CD14+ monocytes from the peripheral blood of three healthy adult donors. These were then cultured in media containing M-CSF for seven days to differentiate the monocytes to macrophages (Fig. 1a). On day 7, we stimulated the macrophages with the Toll-like receptor (TLR) ligands Pam3CSK (which activates TLR2), Lipid A (TLR4), and poly(I:C) (TLR3), and the cytokines TNF*α* and IFN*β* in a time course over ten hours, and performed RNA-seq. There was a high degree of reproducibility between the one female (Donor 2) and two male donors, with correlation coefficients between replicates ranging from 0.940 to 0.984.

We observed that the gene expression programs were highly stimulus-specific. Principal component analysis (PCA) revealed divergent gene expression patterns for the five stimuli (Fig. 1b), and using K-means clustering we identified nine distinct gene expression clusters based on stimulus-specificity (Fig. 1c). As one would predict from the established models of innate immune signaling networks, TNF*α* and Pam3CSK displayed similar patterns in the PCA and heatmap with only subtle differences, for instance in Clusters 5 and 6 where Pam3CSK induced more robust gene expression than TNF*α*. Consistent with the known induction of Type I IFNs by TLR3 signaling, poly(I:C) and IFN*β* also induced similar responses, with the exception of a few genes in Clusters 1 and 3, presumably due to poly(I:C)’s activation of NFκB through TRIF. Lipid A was at the center of the PCA plot and induced virtually all the genes in the heatmap as one would predict, given that TLR4 signaling is known to activate multiple transcription factors through MyD88-dependent and independent pathways.

To further our understanding of the regulatory control of stimulus-specific gene expression programs, we performed an analysis of transcription factor binding motifs in promoters of induced genes (Fig. 1d). Confirming our prior understanding of the signaling networks downstream of PAMPs and cytokines, NFκB motifs were enriched in Clusters 1, 2, 4, 5, and 8, and ISRE motifs were enriched in Clusters 7, 8, and 9. Having validated the stimulus-specificity of gene expression programs in our macrophage system, we next used these transcriptomic phenotypes to understand the effects of Type I vs Type II IFN conditioning on the stimulus-responsiveness of human macrophages.

### Conditioning with Type I or II IFN differentially alters macrophage gene expression responses to stimuli

**Figure 2:**
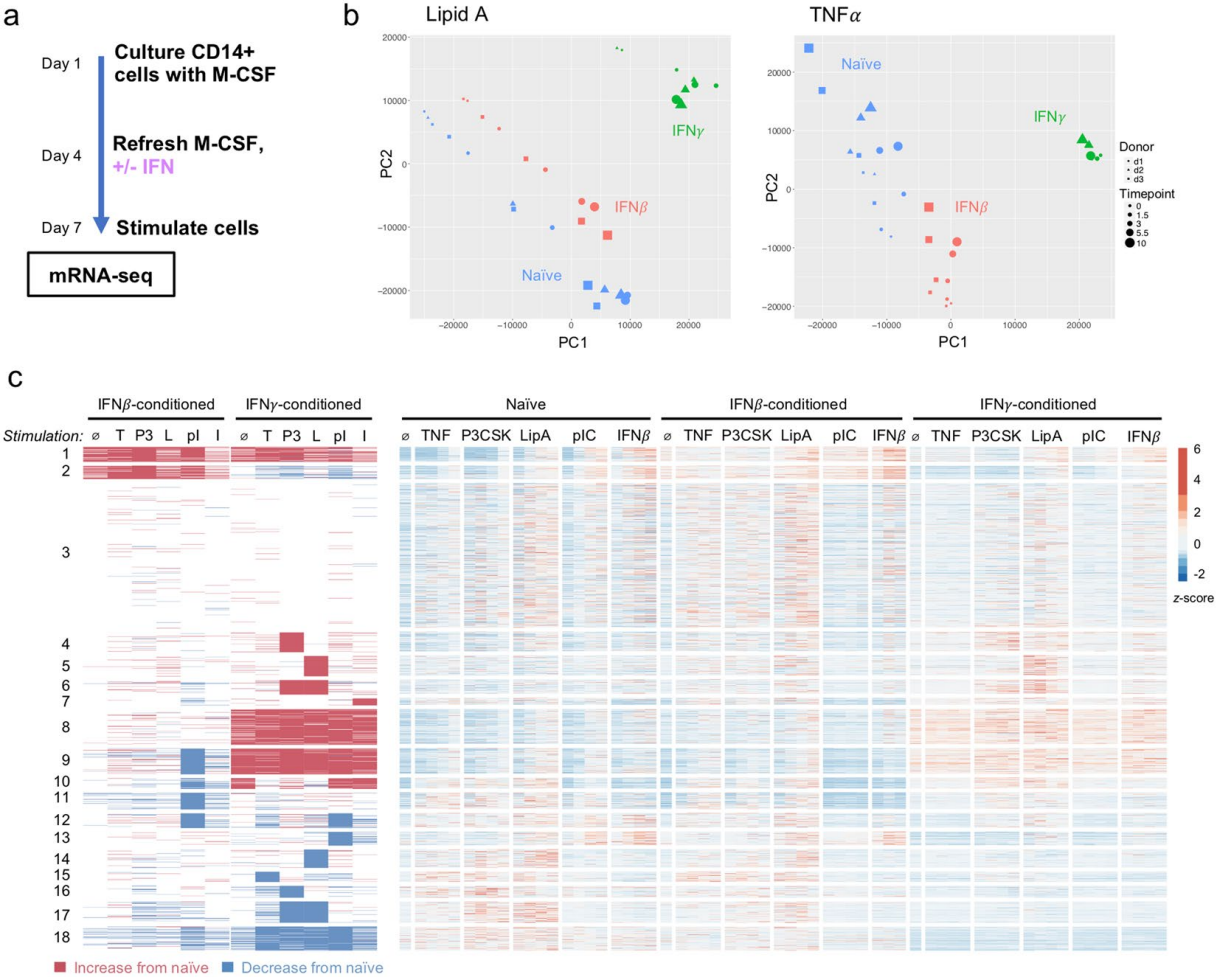
Type I and II IFN have gene-specific and stimulus-specific effects on gene expression. (**a**) Experimental design. IFN*β* (200 U/ml) or IFN*γ* (10 ng/ml) were added on Day 4 of macrophage differentiation, 64 hours prior to stimulation. (**b**) Representative PCA plots for two of the five stimuli illustrating the differential effect of IFN*β* vs IFN*γ* on stimulus-responsive gene expression. (**c**) Master heat map of all conditions. On the right, biological replicates are averaged, and z-scores for 1754 genes are represented. “$\varnothing$ ” denotes unstimulated sample, and each stimulus contains four time points: 1.5, 3, 5.5, and 10 hours. On the left, the same data are represented as fold-change of IFN-conditioned relative to naïve. Red denotes genes where IFN conditioning results in a maximum induction that is 4-fold greater than naïve, blue denotes genes where IFN conditioning results in 4-fold decrease, and white denotes genes where IFN conditioning does not affect expression. Genes are grouped into 18 clusters by the effect of conditioning on stimulus-responsive gene expression.

On day 4 of the M-CSF differentiation process, IFN*β* or IFN*γ* was added and left in the medium through day 7 to condition the macrophages (Fig. 2a). IFN-treated and untreated (“naïve”) macrophages were then stimulated with the same five stimuli on day 7 in a 10-hour time course, and RNA-seq was performed on all samples. Altogether, RNA-seq libraries from 152 samples encompassing three biological replicates, three conditioning regimens, five stimuli, and five time points were analyzed.

Our first observation was that IFN*β* and IFN*γ* had substantial and distinct effects on the basal transcriptomic state of macrophages, with a correlation coefficient of only 0.110 (Fig S1a) at the zero-hour time point prior to the second stimulation. We found that IFN*γ* had a larger effect on basal gene expression than IFN*β*, in agreement with the fact that our widely accepted protocol of using M-CSF for monocyte-to-macrophage differentiation produces macrophages whose basal transcriptome is dependent on tonic Type I IFN signaling^30^. These effects could be visualized by the distance between unstimulated samples in PCA plots of naïve, IFN*β*-, and IFN*γ*-conditioned macrophages (Fig. 2b, S2). Despite the overall discordance, we identified a subset of genes that were concordantly down-regulated. Ontology analysis (Fig S1b) of these genes revealed roles in cell cycle, mitosis, and chromosome organization, suggesting that both IFNs inhibit macrophages from proliferating.

Although there were gene expression differences between Type I and II IFN at the basal state, we hypothesized that many effects of IFN conditioning would only be observed upon second simulation. To address this hypothesis, we developed an analytical workflow to address the complexity of the datasets. We first averaged counts across replicates, collapsed the four time points into a maximum fold-induction for each stimulation condition, and then classified gene expression responses into three categories based on a gene’s expression in the IFN-conditioned stimulation relative to the naïve stimulation using a four-fold threshold to define increase, decrease, or unchanged. We then performed K-means clustering based on this discrete classification system and identified 18 clusters that demonstrated the distinct effects of IFN conditioning on gene expression responses to each stimulus (Fig. 2c, left). 1754 genes were included in the analysis: the 1421 genes that were inducible in naïve macrophages plus an additional 333 genes that met criteria for induction only when conditioned with an IFN (see Supplemental Table 1 for counts).

We found that many genes fit our hypothesis of differential IFN effects that were observable only upon second stimulation. For instance, IFN*γ* and IFN*β* had similar effects on the genes in Cluster 6 at the basal state (“$\varnothing$” column), yet IFN*γ* conditioning potentiated these genes’ response to Pam3CSK and Lipid A whereas IFN*β* conditioning had no effect. In another example, IFN*β* conditioning of the genes in Cluster 10 had no effect on the basal expression but diminished their response to poly(I:C), whereas IFN*γ* conditioning increased both basal expression and their responses to poly(I:C).

To visualize these data without imposed thresholds, we plotted z-scores in a heatmap with the same clusters, also including individual time point information (Fig. 2c, right). We found that the relationships we observed in the thresholded analysis on the left were preserved when visualized as z-scores on the right, though in some instances the thresholded analysis exaggerated the true quantitative effect. Overall, this analysis demonstrated that the differential effects of Type I and II IFN are both gene-specific and stimulus-specific. That is, for a given gene, IFN*β* and IFN*γ* could have opposing effects on its response to one PAMP, but similar effects on its response to another PAMP.

### IFN conditioning potentiates or diminishes the stimulus-responsiveness of genes not induced by IFN alone

We next focused on the genes whose expression was unchanged by IFN alone yet exhibited a potentiated or diminished response to second stimulation when conditioned with IFN. To further explore this group of genes we categorized all treatment conditions into nine categories: first by the effect of IFN treatment alone, i.e. “basal” gene expression, then by their conditioned response to stimulation compared to naïve (Fig S3a,b). For this analysis, we used a two-fold threshold to more stringently identify genes that had “no change” in the basal state. We then used a four-fold threshold to categorize the stimulus-responsiveness of the conditioned macrophages as “unaffected,” “potentiated,” or “diminished” compared to naïve.

**Figure 3:**
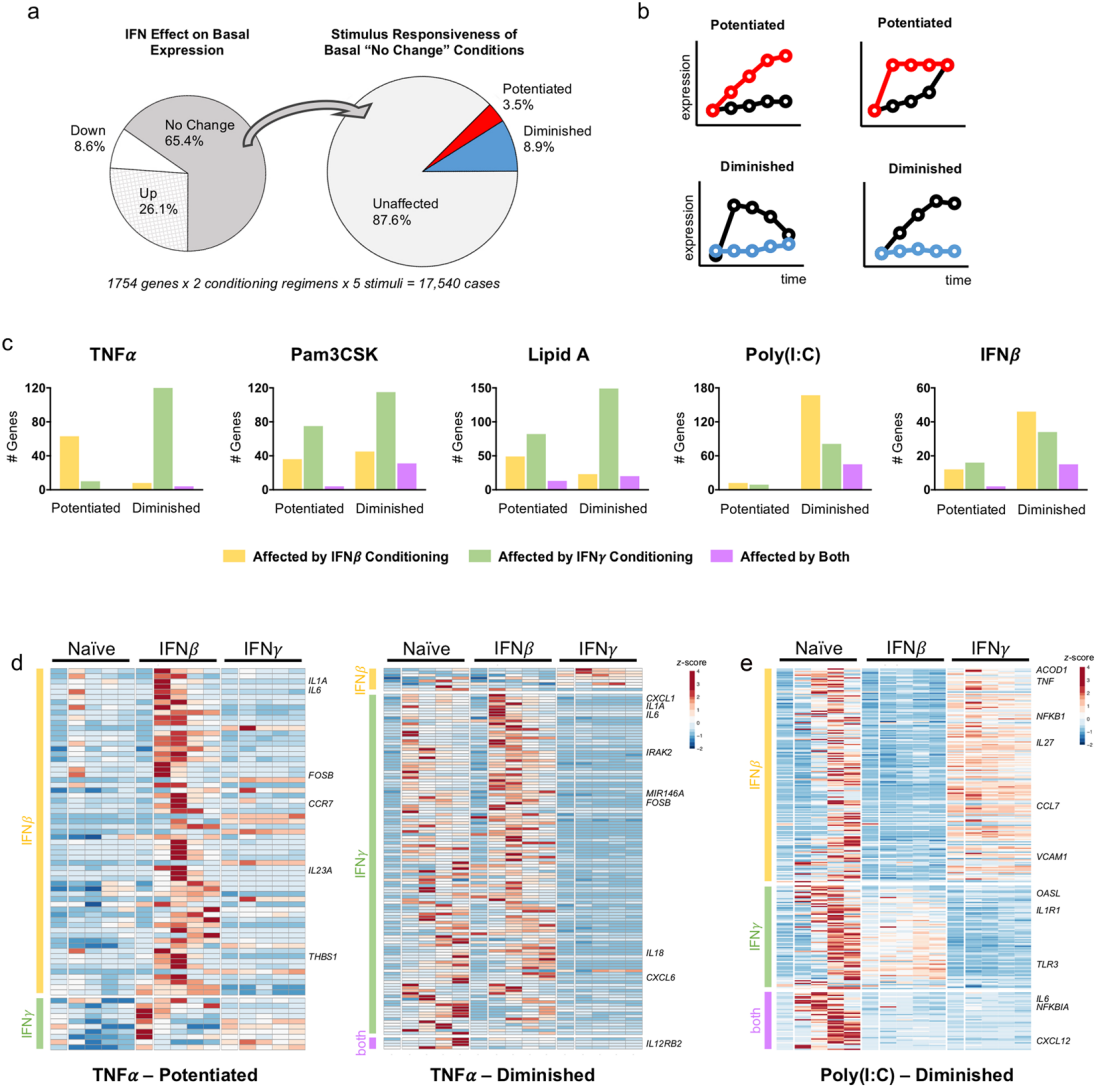
IFN conditioning potentiates or diminishes the stimulus-responsiveness of genes not induced by IFN alone. (**a**) Left: effect of either IFN prior to second stimulation (two-fold threshold). Right: of the genes not changed by IFN alone, the distribution of genes with potentiated or diminished response to a stimulus. (**b**) Cartoons illustrating the categories in right-hand pie chart of (**a**) Black = naïve, Color = conditioned. (**c**) Number of genes in each category, separated by stimulus. (**d**) TNFα-inducible genes that are unaffected by IFN alone but have a potentiated (left) or diminished (right) response after IFN conditioning, clustered by whether the criteria are met in IFN*β* conditioning, IFN*γ* conditioning, or both. (**e**) Genes that are unaffected by IFN alone but have a diminished response to poly(I:C).

For each of the 1,754 inducible genes, ten cases were analyzed: two conditioning regimens and five stimulation conditions. At the basal state, we found that 65.4% of cases fell within a range of two-fold change and were considered “no change” by IFN treatment alone (Fig. 3a). Of these cases that were unchanged, we found that 12.4% were nonetheless potentiated or diminished in their response to a second stimulation, with 8.9% of cases showing a diminished response, and 3.5% showing a potentiated response. Altogether 713 genes had responses to one or more stimuli that were potentiated or diminished by IFN conditioning.

We performed Ingenuity pathway analysis on these genes and found that canonical pathways related to immune functions were significantly overrepresented (Fig S3c). For many of these pathways, IFN*β* and IFN*γ* conditioning had a similar effect, frequently diminishing gene expression responses. For instance, IL-10 signaling and granulocyte adhesion and diapedesis were overrepresented in the genes diminished by conditioning with either IFN. On the other hand, some pathways were potentiated by IFN*γ* but diminished by IFN*β* conditioning, such as dendritic cell maturation. This pathway analysis provided a general sense that many relevant immunological pathways were affected by IFN conditioning, but did not identify any unifying functional themes.

To obtain a more detailed understanding of the “no change/potentiated” and “no change/diminished” gene responses, we organized our analysis in a stimulus-centric manner (Fig. 3c, S4). We found that macrophage responses to TNF were dramatically different in Type I vs Type II IFN conditioning. IFN*γ* potentiated only 10 genes but diminished 120 genes, while IFN*β* potentiated 63 genes and diminished only 8 genes. This included many genes with well-defined roles in immune responses. For instance, IFN*β* potentiated *IL1A*, *IL6*, and *CCR7*, while IFN*γ* diminished the TNF response of *IL1A*, *IL6*, *IL18*, and *CXCL1* (Fig. 3d, full gene list in Supplemental Table 2). There were also very few genes that were potentiated or diminished by both IFNs. These results suggest that, for genes not directly induced by IFN, Type I and II IFNs have opposing and non-overlapping effects on macrophage responses to TNF.

Pam3CSK and Lipid A responses were also significantly affected by IFN conditioning (Fig. 3c). In contrast to the TNF responses, one could not make a generalized statement about the direction of the effects of IFN*β* or IFN*γ* on Pam3CSK and Lipid A responses. Instead, both IFNs are able to potentiate and diminish gene expression responses. A key observation, therefore, is that the effects of IFN conditioning on TLR2 and TLR4 responses are gene-specific. For instance, IFN*γ* had opposing effects on two chemokines that are reported to both recruit neutrophils^31^: in response to Pam3CSK, *CXCL3* was potentiated and *CXCL6* was diminished by IFN*γ* conditioning (Supplemental Table 2).

IFN conditioning had a striking effect on poly(I:C) and IFN*β* responses (Fig. 3c,e). Here, the vast majority of effects were of diminished gene expression response, demonstrating that both IFNs, classically produced in the context of viral infection, can tolerize macrophages and diminish their subsequent response to the viral dsRNA-mimetic poly(I:C) and additional antiviral cytokines. Importantly, however, only a minority of these genes were affected by both IFN*β* and IFN*γ*. In fact, many genes whose poly(I:C)-responsiveness was diminished by IFN*β* were directly upregulated by IFN*γ* treatment (Fig. 3e), suggesting that IFN*γ* directly induces a subset of the poly(I:C) gene expression program that is inhibited by IFN*β* conditioning. The reverse is also true – many poly(I:C)-responsive genes that are also induced by IFN*β* are inhibited by IFN*γ* conditioning.

### IFN conditioning differentially alters cytokine and chemokine expression in a stimulus-specific manner

**Figure 4:**
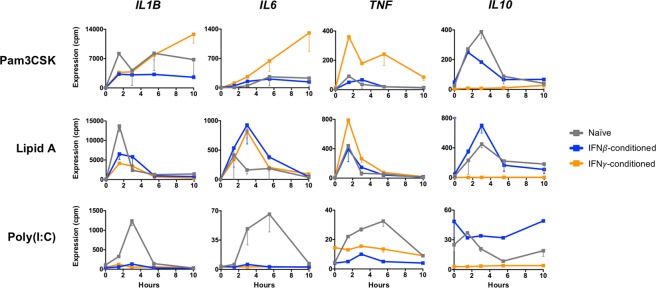
Effects of IFN*β* vs IFN*γ* conditioning on inflammatory and anti-inflammatory cytokines.

Many of the genes we identified in the analysis of “no change/potentiated” and “no change/diminished” groups were cytokines and chemokines. One widely accepted model of Type I and II IFN contends that IFN*γ* is pro-inflammatory while IFN*β* is anti-inflammatory^16,17^. We therefore assessed the effect of IFN conditioning on transcript levels of the well-established inflammatory cytokines IL1*β*, IL6, and TNF and the anti-inflammatory cytokine IL10 in response to TLR stimulation (Fig. 4). We found that some conditions were consistent with the proposed model, such as *IL6* and *TNF* in responses to Pam3CSK, where IFN*γ* primed macrophages for potentiated gene expression. We also saw that IFN*γ* conditioning dramatically suppressed *IL10* induction, while IFN*β* preserved the expression of this anti-inflammatory cytokine. However, there were also conditions where the gene expression pattern did not conform to the proposed model. For instance, conditioning with either IFN completely abrogated the expression of *IL1B* and *IL6* in response to poly(I:C). Both IFNs also had parallel effects on potentiating *IL6* responses to Lipid A. These stimulus-specific effects of IFN*β* and IFN*γ* challenge the idea that IFN*γ* is strictly pro-inflammatory and IFN*β* anti-inflammatory.

Our genome-wide analysis also suggested that exposure to Type I or II IFN modulated subsequent chemokine production. We grouped chemokines by the primary cell type recruited^31^ and assessed the effect of IFN conditioning on gene expression after TLR stimulation (Fig S5). Even in naïve macrophages, without IFN conditioning, we observed stimulus-specific patterns of chemokine expression. TLR3 stimulation, for instance, induced more expression of lymphocyte and monocyte-recruiting chemokines than neutrophil-recruiting chemokines. Exposure to either IFN had relatively little effect on the basal expression of chemokines prior to second stimulus. However, in response to TLR2 or TLR4 stimulation, conditioning with IFN*γ* tended to enhance lymphocyte-recruiting chemokines and diminish chemokines involved in recruitment of monocytes and neutrophils. The majority of chemokines, however, had specific effects depending on the type of IFN and the type of PAMP. For instance, expression of *CXCL8* was potentiated by IFN*γ* but slightly diminished by IFN*β* for TLR2 and TLR3 stimulation and unaffected by either IFN for TLR4 stimulation, again illustrating that the effects of IFN conditioning are gene-specific and stimulus-specific.

### IFN conditioning differentially affects signaling networks

Having established that IFN conditioning has stimulus-specific effects on a genome-wide level as well as on relevant single genes, we next explored potential mechanisms for these phenomena. We considered that IFN conditioning may affect the strength of stimulus-responsive signaling networks and the chromatin environment of target genes, which together may result in stimulus- and gene-specific potentiation or reduction in gene activation.

**Figure 5:**
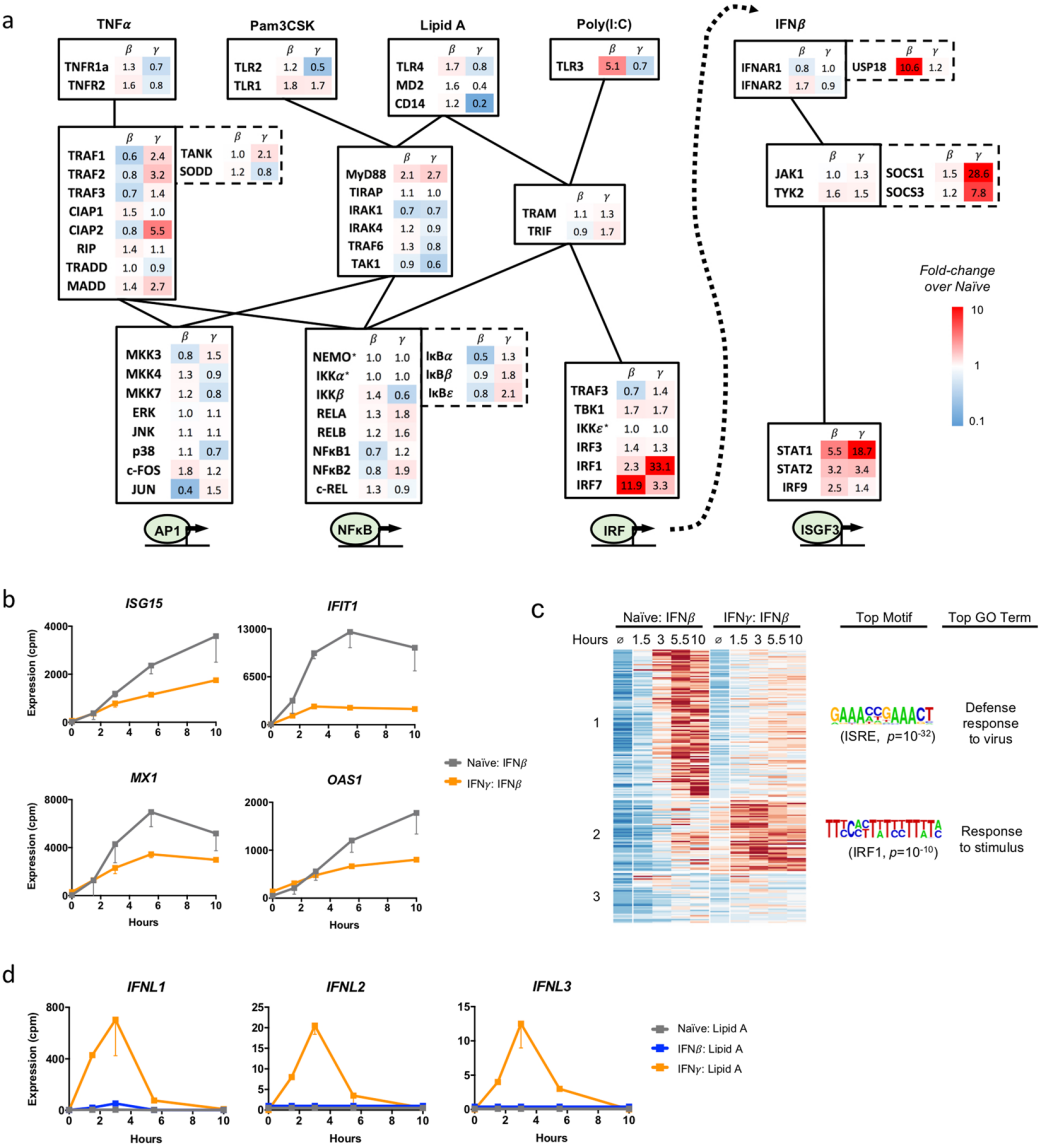
IFN conditioning alters innate immune signaling networks. (**a**) The effects of IFN conditioning on genes that participate in innate immune signaling are shown as fold-change over naïve expression. Genes are arranged in their known signaling networks, and negative regulators appear in dashed boxes. Asterisks (*) denotes genes with zero counts in all samples. (**b**) Conditioning with IFN*γ* diminishes responsiveness to IFN*β* stimulation for four well-established IFN*β* inducible genes, consistent with IFN*γ* upregulation of SOCS1 and SOCS3. (**c**) 205 IFN*β*-inducible genes (>10-fold induction in naïve macrophages) are plotted in a heatmap and clustered by effect of IFN*γ* conditioning. Top results of transcription factor motif and gene ontology analyses are shown for the clusters affected by IFN*γ* conditioning. (**d**) Conditioning with IFN*γ* potentiates induction of IFNλ genes in response to Lipid A, consistent with IFN*γ* upregulation of IRF1.

To examine whether conditioning with IFN might affect PAMP and cytokine-responsive signaling networks, we assessed the impact of IFN*β* and IFN*γ* conditioning on the basal expression of genes that encode the transcriptional networks downstream of TNFR, TLR2, TLR4, TLR3, and IFNAR (Fig. 5a). We found a number of substantial changes in expression of both positive and negative regulators. For instance, *TLR3* expression was increased 5.1-fold and *IRF7* was increased 11.9-fold by IFN*β* conditioning. The changes in TLR3 and IRF7 would predict increased responses to poly(I:C) when conditioned with IFN*β*, but expression of *USP18*, a key negative regulator of IFNAR^32^ was also dramatically increased, perhaps mitigating the poly(I:C) response.

The effect of IFN*γ* conditioning on IRF and ISGF3 signaling was particularly striking. IFN*γ* conditioning resulted in an 18.7-fold increase in *STAT1*, a 3.4-fold increase in *STAT2*, and a 1.4-fold increase in *IRF9*. These three proteins form the ISGF3 transcription factor downstream of IFNAR signaling, so one might anticipate that IFN*γ* potentiates IFN*β* signaling. However, we also observed a 28.6-fold increase of SOCS1 and a 7.8-fold increase of SOCS3. These suppressors of cytokine signaling inhibit IFNAR signaling by blocking and dephosphorylating JAKs^33^.

Given the potentially conflicting activities of STAT and SOCS upregulation as well as the findings in Fig. 3e, we explored at a more granular level whether IFN*γ* diminishes or potentiates IFN*β*-responsive genes. We found that expression of the well-established IFN*β* stimulated genes *ISG15*, *IFIT1*, *MX1*, and *OAS1* were diminished by IFN*γ* conditioning (Fig. 5b). To address this question in an unbiased manner, we defined IFN*β* responsive genes as any gene that was induced 10-fold or higher by IFN*β* in naïve macrophages. We then performed K-means clustering of these 205 genes (Fig. 5c) and found that 113 of them behaved similarly, with relatively unchanged basal expression but diminished responsiveness to IFN*β* when conditioned with IFN*γ* (Cluster 1). However, we also identified a second cluster of genes in which IFN*γ* conditioning had the opposite effect of increasing expression both at basal and in response to IFN*β* and a third cluster in which IFN*γ* had no effect.

To further understand the differences between the genes in these three clusters, we performed transcription factor motif and gene ontology (GO) analysis. The top GO term for the genes in Cluster 1 was “Defense response to virus,” and the top transcription factor binding motif was for ISRE, the canonical binding motif for ISGF3. This suggested that the genes diminished by IFN*γ* conditioning were classical, antiviral IFN*β* stimulated genes under the control of ISGF3, including *ISG15, IFIT1, MX1*, and *OAS1*, whose activity may be diminished by the induction of SOCS proteins. In contrast, the top GO term for Cluster 2 was a generic “response to stimulus,” and the top transcription factor motif was for IRF1. These results suggested that the genes potentiated or unchanged by IFN*γ* conditioning are functionally different from those in Cluster 1 and that they are co-regulated by different transcription factors. The presence of IRF1 binding motifs in the promoters of Cluster 2 is particularly interesting given that *IRF1* expression was upregulated 33-fold by IFN*γ* conditioning. This supports the possibility of crosstalk between IFNAR signaling and IRF1 that synergistically activates a subset of IFN*β* stimulated genes.

IRF1 is also known to play a key role in the regulation of Type III IFN (IFN*λ*) expression^34,35^. Indeed, we observed that IFN*γ* conditioning dramatically up-regulated the IFN*λ* genes *IFNL1*, *IFNL2*, and *IFNL3* in response to Lipid A (Fig. 5d). In the naïve condition these Type III IFN genes are not induced at all by any stimulus. Interestingly, IFN*γ*’s ability to potentiate IFN*λ* expression, which was reproducible between biological replicates, was specific only to Lipid A. Pam3CSK, poly(I:C), TNF, and IFN*β* stimulation did not induce IFN*λ* expression in any condition. This suggests a complex regulation of IFN*λ* expression involving IRF1 but possibly also requiring other factors that are only activated upon TLR4 stimulation.

### IFN conditioning differentially affects chromatin landscape

Whereas changes in signaling networks are likely to result in stimulus-specificity, changes in the epigenome, with gains and losses of accessible enhancers, may be a mechanism for the gene-specific effects of cytokine conditioning. We therefore sought to define the effects of IFN*β* and IFN*γ* on the chromatin landscape by measuring DNA accessibility. On Day 7, prior to secondary stimulation, we performed ATAC-seq on naïve, IFN*β*-conditioned, and IFN*γ*-conditioned macrophages in biological replicate. We found that conditioning with either IFN resulted in differential ATAC-seq signals corresponding to gains and losses of transposase-accessible sites. IFN*γ* conditioning resulted in 4.5-times more differential peaks than IFN*β* (9562 versus 2085), and 705 of these peaks were overlapping.

**Figure 6:**
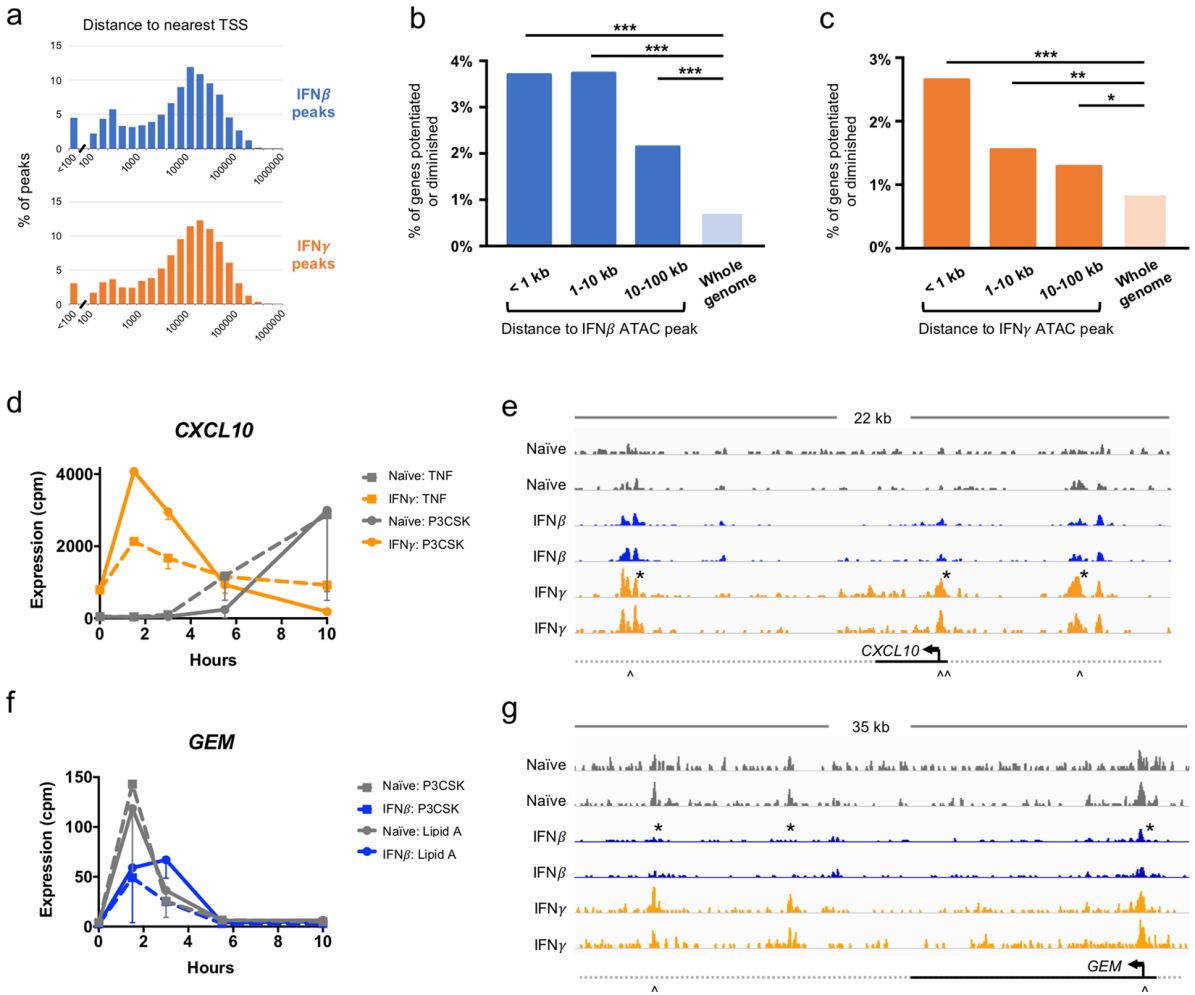
IFN conditioning affects chromatin landscape. (**a**) Distribution of the distance from differential ATAC-seq peaks to the nearest TSS. (**b**,**c**) Relationship of differential ATAC-seq peaks to gene expression. Based on RNA-seq data, 419 and 493 genes were categorized as potentiated or diminished by IFN*β* and IFN*γ*, respectively. Regions near ATAC-seq peaks are enriched for these genes. (* = *p < *0.01, ** = *p* < 0.001, *** = *p* < 0.0001) (**d**) Example of gene potentiated by IFN*γ*, with more rapid response to TNF and Pam3CSK when primed. (**e**) ATAC-seq tracks at CXCL10 locus in replicate, showing three peaks that are gained by IFN*γ* conditioning. Asterisks (*) denote differential peaks, and arrows (^) denote NFκB motifs. (**f**) Example of gene diminished by IFN*β*, with more delayed and decreased response to Pam3CSK and Lipid A after IFN*β* conditioning. (**g**) ATAC-seq tracks at GEM locus show three peaks that are lost with IFN*β* conditioning. Asterisks (*) denote differential peaks, and arrows (^) denote NFκB motifs.

To assess the biological relevance of these ATAC-seq peaks, we surveyed their genomic distribution relative to transcription start sites (TSSs). We found that both IFN*β* and IFN*γ* peaks were distributed near TSSs for annotated genes (Fig. 6a), with 95% of both IFN*β* and IFN*γ* peaks falling within 100 kilobases (kb) of a TSS. Additionally, 19% of IFN*β* peaks and 12% of IFN*γ* peaks were found in potential promoter regions, within 1 kb of a TSS. The proximity of ATAC-seq peaks to gene TSSs suggested that these gains and losses in chromatin accessibility were not randomly distributed in the genome but may correspond to *cis-*acting gene regulatory elements.

Next, we investigated whether there was a correlation between these ATAC-seq peaks and our gene expression data. We utilized the previously described nine categories of gene expression responses (Fig S3a) and focused on four conditions relevant to the ATAC-seq analysis. We inferred that if an enhancer was gained or lost by IFN conditioning, the resultant gene expression response to stimulus would fall into one of four categories: “no change/potentiated,” “no change/diminished,” “up/potentiated,” or “down/diminished.” 419 and 493 genes fell into these categories for one or more stimuli for IFN*β* and IFN*γ*, respectively.

We then asked whether the regions around ATAC-seq peaks were enriched for these potentiated or diminished genes. We found that, compared to the whole genome, the regions around ATAC-seq differential peaks were significantly enriched, with a trend towards greater enrichment at the most proximal genes (Fig. 6b,c). Genomic regions within 1 kb of a differential ATAC-seq peak were enriched 5.1-fold in IFN*β* conditioning and 3.1-fold in IFN*γ* conditioning. Interestingly, despite the enrichment near ATAC-seq peaks, the majority of potentiated or diminished genes still fell in regions of the genome that are not near an ATAC-seq peak. This may reflect the difficulty in relating enhancer function to a particular gene, or may suggest that other mechanisms not assayable by ATAC-seq are responsible for their gene expression.

To corroborate our genome-wide analyses, we investigated single gene examples where differential ATAC-seq peaks were correlated with gene expression changes. *CXCL10* is an NFκB target gene and is induced by TNF and Pam3CSK in naïve macrophages at late time points (Fig. 6d). When conditioned with IFN*γ*, *CXCL10* was more highly expressed at basal steady-state, and its response to TNF and Pam3CSK was much more rapid, peaking at (or before) 1.5 hours. ATAC-seq analysis revealed three peaks near *CXCL10* that were gained in IFN*γ* conditions, one at the promoter, one 5.1 kb upstream, and one 11.4 kb downstream of the TSS (Fig. 6e). Each of these peaks contained at least one NFκB binding motif, strongly suggesting a mechanism where IFN*γ* renders these latent enhancers more accessible and *CXCL10* primed to respond more rapidly to NFκB-activating stimuli.

Similarly, the expression of *GEM* in response to TNF and Pam3CSK is diminished by conditioning with IFN*β* (Fig. 6f). *GEM* is also an NFκB target gene, and IFN*β* conditioning results in the loss of three ATAC-seq peaks, one in the promoter, and two downstream (Fig. 6g). The ATAC-seq peaks at the promoter and 17.8 kb downstream of the TSS contain NFκB binding motifs, suggesting a mechanism where IFN*β* conditioning results in silencing of previously active NFκB enhancers. These examples and our genome-wide analysis both imply that the differential peaks identified by ATAC-seq were biologically relevant and co-localized with genes whose expression is potentiated or diminished by IFN conditioning.

## Discussion

Here we have reported the results of an unbiased, genome-wide analysis of the effects of Type I vs Type II IFN conditioning on the stimulus-responsive gene expression patterns of primary human macrophages. An essential feature of this study was the use of a sequential conditioning and stimulation approach. By examining not only the direct consequences of IFN treatment but focusing on subsequent responses to pathogen-associated stimuli, we gained novel insight into the gene-specific and stimulus-specific effects of Type I and II IFN. Our approach enabled us to identify subtle but important differences between Type I and II IFN, including their opposing effects on TNF-inducible genes, the negative regulation of antiviral Type I IFN-stimulated genes by IFN*γ* conditioning, and the potentiation of Type III IFN genes by IFN*γ*. We found that the IFNs modulate macrophage function in a highly nuanced manner that is not uniformly pro- or anti-inflammatory. These immune regulatory functions of IFN could not be gleaned by examining only direct IFN-induced gene expression programs, thus highlighting the importance of the sequential conditioning-stimulation approach.

One of our most notable findings was that IFN*γ* and IFN*β* have opposing effects on macrophage responses to TNF. IFN*γ* substantially diminished TNF responses, with 120 genes falling into our “no-change/diminished” category. This was a surprising finding given that IFN*γ*-conditioned macrophages are thought to be more pro-inflammatory, and TNF is a prototypical inflammatory cytokine. The mechanism of this phenomenon is uncertain; our analysis of signaling networks was unrevealing in this respect as a number of factors in the TNF signaling pathway such as *TRAF1*, *TRAF2*, *CIAP2*, and *MADD* were actually increased by IFN*γ* (Fig. 5a). One possibility, since TNF is an IFN*γ* target gene^36^ and is upregulated three-fold by IFN*γ* in our dataset, is that IFN*γ* conditioning leads to an increase in tonic TNF which tolerizes macrophages to additional TNF^37^. Together with a recent study showing that IFN*γ* restricts the induction of some inflammatory cytokines in response to TLR4 stimulation^38^, our data challenges the generalization that IFN*γ* makes macrophages more inflammatory.

In contrast to IFN*γ*, IFN*β* conditioning generally potentiated macrophage responses to TNF. This is in agreement with previous observations that TNF and IFN*β* synergistically induce gene expression, possibly through a STAT1-independent ISGF3 complex^18,39^. Additionally, a recent study showed that conditioning with both IFN*β* and TNF potentiates responses to LPS compared to TNF conditioning alone^40^. Our data thus provides additional support for the model where TNF and IFN*β* can cooperatively regulate macrophage gene expression and extends this notion to describe that prior IFNβ exposure enables TNF to potently activate a new set of target genes. The contrasting effects of Type I and II IFN on TNF gene expression responses are highly relevant for our understanding of immune responses *in vivo*, where all three cytokines could be present simultaneously, and cautions against the simple characterization of Type I IFN as being anti-inflammatory. The regulatory logic controlling the interplay of these key cytokines deserves further attention.

While IFN*β* and IFN*γ* had opposing effects on TNF responses, conversely, we found that they had similar effects on poly(I:C) and IFN*β* responses (Figs 3f,g, 5b,c). Both IFNs diminished these gene expression programs, demonstrating the importance of negative feedback loops in the IFN regulatory system. Indeed, a number of negative regulators, including SOCS1, SOCS3, and USP18^32,33,41^, are upregulated by IFN conditioning. Physiologically, it makes sense that prolonged Type I IFN stimulation tolerizes cells to subsequent Type I IFN stimulation. However, the repressive effect of IFN*γ* conditioning on subsequent response to Type I IFN stimulation is noteworthy. It suggests that when IFN*γ* is dominant or pre-existent, as in an intracellular bacterial infection, there is a functional advantage to silencing the antiviral portion of the IFN*β* transcriptome, which may be superfluous in this context. Together with prior work showing a similar inhibition in the reverse direction where IFN*β* inhibits IFN*γ* gene expression^24^, one can begin to conclude that Type I and II IFN gene expression programs are cross-repressive when macrophages are conditioned in one and then exposed to the other.

Another intriguing and novel finding was that IFN*γ* conditioning dramatically potentiated induction of Type III IFN genes in response to Lipid A. This result was particularly noteworthy for its specificity for TLR4 and not TLR2 or TLR3 stimulation. That TLR4, typically a receptor for bacterial PAMPs and host-derived DAMPs, results in IFNλ expression under IFN*γ* conditions suggests that IFNλ might play additional roles beyond its described antiviral function at epithelial barriers^42^. The specificity for TLR4 stimulation also raises questions about the mechanisms controlling IFN*λ* expression. The *IFNL1* promoter shares many features with *IFNB*, and it is thought that NFκB and IRFs, particularly IRF1, cooperatively induce gene expression of IFNλ genes^34,35,42^. IRF1 is highly upregulated by IFN*γ* conditioning, suggesting a potential mechanism for IFN*γ*’s potentiation of IFNλ induction. Additionally, a recent study found that in human monocyte-derived dendritic cells, *IFNL1* expression was p38 MAPK-dependent^43^. This may provide an explanation for TLR4-specificity, but further studies into the mechanisms of IFNλ regulation are clearly warranted.

Many prior studies have implicated Type I and II IFNs in regulating expression of cytokines and chemokines, with wide-ranging clinical implications such as viral-bacterial co-infections, host response to leprosy, response to DAMPs, and connections to autoimmunity. The contribution of our study to this field is to show that chemokines and cytokine production is modulated by IFN conditioning in a stimulus-specific manner. It is overly simplistic, for instance, to say IFN*γ* potentiates inflammatory cytokines when poly(I:C) stimulation actually induces much less *IL1B* and *IL6* when conditioned with IFN*γ*. Here we have addressed specificity for synthetic TLR stimuli. By extension, our findings imply that *in vivo* there will also be specificity for different pathogens. For example, although IFN*β* increases susceptibility to bacterial pneumonia following influenza infection due to an impairment of neutrophil recruitment and IL17-mediated immunity^44,45^, these mechanisms may not hold true for fungal superinfections, which activate the immune system through different receptors.

In this study, we also explored potential mechanisms of context-specific responses. We found that Type I and II IFNs altered both the basal signaling network and the chromatin accessibility of cells and described examples of potentiated and diminished gene expression that may be a consequence of these perturbations. These findings support prior models that posit that signaling networks encode stimulus information into the activity of transcriptional effectors, and epigenetic states decode that information into a context-dependent, stimulus-specific gene-expression program and biological response^46^. We show here that both the encoding and decoding steps are affected by cytokine context. It is likely that stimulus-specificity is driven by alterations in signaling networks while gene-specific differences are a result of epigenetic transcriptional control, and the interdependent relationship between the two is what gives rise to highly tunable, context-specific immune responses.

Indeed, *in vivo*, macrophages are simultaneously exposed to multiple cytokines that may also vary in dose and duration of exposure. The space of possible conditions is in fact too large to systematically probe experimentally, and therefore one goal of studies such as the present, that characterize well-defined points within this space, is to catalyze the development of data-driven and mechanistic computational models (e.g. Cheng *et al*.)^47^ to fill in the regulatory landscape. Such models may then also provide analytical frameworks without the use of intuitive thresholds we have employed here to analyze high-complexity data and define categories such as expressed, inducible, potentiated, or diminished genes. However, what constitutes a feasible strategy for developing such models that account for condition- or context-dependent states of signaling systems and epigenomic responsiveness requires further theoretical work before they can be deployed. The present dataset and the scope, range, and granularity of the observations should prove useful in guiding such computational modeling investigations.

## Materials and Methods

### Macrophage cell culture

Whole blood was obtained from healthy donors with written informed consent prior to inclusion in the study according to protocol #11-001274 approved by the UCLA Institutional Review Board. Peripheral blood mononuclear cells (PBMCs) were isolated using Ficoll (GE Healthcare, Piscataway, NJ) gradient centrifugation. Monocytes were purified by positive selection of CD14+ cells using MACS CD14 microbeads (Miltenyi Biotec, Cologne, Germany) from PBMCs according to manufacturer’s instructions. Macrophages were derived from CD14+ positively-selected monocytes by differentiation for seven days in RPMI (Thermo Fisher Scientific, Waltham, MA) with 10% fetal bovine serum (Omega Scientific, Tarzana, CA), glutamine, and penicillin-streptomycin supplemented with 50 ng/ml recombinant human M-CSF (CHO-derived, R&D Systems, Minneapolis, MN) at a concentration of 0.5 × 10^6^ monocytes/ml in 24-well plates (Corning Inc., Corning, NY). On day four, 64 hours prior to stimulation, a 1/5^th^ volume of fresh medium was added containing conditioning cytokines 10 ng/ml IFN*γ* (BD Biosciences, La Jolla, CA) or 200 U/ml IFN*β* (PBL Assay Science, Piscataway, NJ). On Day 4 M-CSF was also refreshed by adding an extra 25 ng/ml (final concentration) on top of any exhausted M-CSF.

### Stimulation and RNA preparation

On day 7, a 1/6^th^ volume of fresh medium with stimuli were added to the following final concentrations: 100 ng/ml Lipid A (InvivoGen), 5 ng/ml TNFα (BD Biosciences), 100 ng/ml Pam3CSK (InvivoGen, San Diego, CA), 20 μg/ml poly(I:C) (InvivoGen), 200 U/ml IFN*β* (PBL Assay Science). Cells were collected at 1.5, 3, 5.5, and 10 hours post stimulation by lysis with TRIzol reagent (Life Technologies, Carlsbad, CA). Total RNA was purified with DIRECTzol kit (Zymo Research, Irvine, CA) according to manufacturer’s instructions.

### Next generation sequencing

For RNA-seq, strand-specific libraries were generated from 500 ng total RNA using KAPA Stranded mRNA-seq Library Preparation kit (KAPA Biosystems, Wilmington, MA) according to the manufacturer’s instructions. Resulting cDNA libraries were single-end sequenced with a length of 50 bp on an Illumina HiSeq 2000 (Illumina, San Diego, CA).

ATAC-seq libraries were prepared as previously described^48^. Briefly, cells were dissociated with Accutase (Thermo Fisher Scientific), and 50,000 cells were used to prepare nuclei. Cell membrane was lysed using cold lysis buffer (10 mM Tris-HCl pH7.5, 3 mM MgCl2, 10 mM NaCl and 0.1% IGEPAL CA-630). Nuclei were pelleted by centrifugation for 10 minutes at 500 × *g*, and suspended in the transposase reaction mixture (25 µl of 2X TD Buffer (Illumina), 2.5 µl of TD Enzyme 1 (Illumina), and 22.5 µl of nuclease-free water). The transposase reaction was performed for 30 minutes at 37 °C in a thermomixer shaker. Then, fragmented DNA in the reaction was purified using MinElute PCR purification kit (QIAGEN, Hilden, Germany). The purified DNA fragments were amplified by PCR to obtain ATAC-seq libraries with Illumina Nextera sequencing primers. The libraries were purified using MinElute PCR purification kit (QIAGEN) and quantified using KAPA Library Quantification Kit (KAPA Biosystems). The libraries were single-end sequenced with a length of 50 bp on an Illumina HiSeq 2500.

All sequencing data was deposited in the National Center for Biotechnology Information (NCBI) Sequencing Read Archive (SRA) and is publicly available under experiment numbers SRP145599 (RNA-seq) and SRP145626 (ATAC-seq).

### Global RNA-seq analysis

The low quality 3′ ends of reads were trimmed (cutoff q = 30), and remaining adapters sequences were removed using cutadapt^49^. Reads were aligned to the hg19 genome build with STAR^50^ with the following options: --outFilterMultimapNmax 20, --alignSJoverhangMin 8, --alignSJDBoverhangMin 1, --outFilterMismatchNmax 999, --outFilterMismatchNoverLmax 0.04, --alignIntronMin 20, and --alignIntronMax 1000000 --seedSearchStartLmax 30. Only uniquely mapped reads with a mapping quality ≥30 were kept for further analysis, using samtools. Read counts were normalized for library size and transcript length by conversion to CPM and RPKM. Genes below an expression threshold of 4 RPKM in all samples were excluded from downstream analysis. Biological replicates of “unstimulated” samples were averaged and considered to be the zero-hour time point or basal expression. The zero-hour data were then placed in a log2-transformed bimodal distribution using the Mix-Tools Package^51^. The first equivalent overlap of the two distributions was 2.2 CPMs, and this pseudo-count was added to all expressed genes. Induced genes were defined as those with a 4-fold increase over basal by any stimulation with FDR threshold of 0.01 calculated with edgeR^52^. Principle components were calculated with the prcomp package^53^ and plotted with ggplots^54^. K-means clustering was performed with the mclust package^55^ with spherical clustering and constant shape and orientation, and the choice of number of clusters was based on the plateau of logliklihood scores. The linear z-score transformation of the CPM values across all samples were plotted as heatmaps using heatmap2 and pheatmap packages^56^.

### Thresholds for Nine-Category analysis

The effect of conditioning on basal gene expression (“up,” “down,” or “no-change”) was determined by calculating fold change of the IFN-conditioned basal over naïve basal, with a threshold of two-fold and FDR of 0.01. Next, the effect of conditioning on inducible gene expression was determined. For cases of “no-change” at basal the direct fold-change was calculated at each time point as the change of conditioned-stimulated over naïve-stimulated gene expression. For each case, the greatest absolute change across time-points was used to categorize the effect of conditioning on inducible gene expression, using a threshold of four-fold and FDR of 0.01, as “potentiated,” “diminished,” or “unaffected.” For cases of “up” or “down” at basal the change in fold change was calculated at each time point to increase the stringency of the analysis for genes already differentially expressed at the basal state. For each case, the greatest change in fold-change across time-points was then used to categorize the effect of conditioning on inducible gene expression, using a threshold of four-fold and FDR of 0.01, as “potentiated,” “diminished,” or “unaffected.” Altogether this yielded nine categories: three basal categories, each with three inducibility subcategories (Fig S3).

### Ingenuity Pathway Analysis

Gene lists falling into “no change/diminished” and “down/diminished” or “no change/potentiated” and “up/potentiated” categories from the Nine Category analysis (above) were uploaded to the Ingenuity Pathway Analysis (IPA) tool (QIAGEN). Default settings were used to obtain enrichment scores for canonical pathways, and *p-*values were calculated by Fisher’s exact test. Hierarchical clustering was performed using default IPA settings.

### Transcription factor motif and Gene Ontology analysis

Transcription factor motif analysis was performed using HOMER^57^ with JASPAR matrices for known NFκB and ISRE motifs to derive *p*-values for overrepresentation of these motifs within a defined promoter region of −600 bp to +50 bp. Search options included absolute match length of 8, 10, 12, 14, or 16 bp; allowed mismatch of 4 bp; and all expressed genes as the background for control. Gene ontology (GO) analysis was performed using the PANTHER database with entire human genome as background^58^.

### ATAC-seq analysis

ATAC-seq reads were aligned to the hg38 genome build using bowtie2 with default parameters except --very-sensitive and --non-deterministic options and filtered based on mapping score (MAPQ ≥ 30) by Samtools version 1.3.1^59^. Duplicated reads were removed using Picard MarkDuplicates (version picard-tools-2.1.0). MACS2 version 2.1.0 was used to identify peaks for each sample individually with default settings except FDR of 0.01^60^. These peaks were merged to generate a single reference peak file, and the number of reads that fell into each peak was counted using bedtools multicov^61^. DESeq2 was used to normalize and identify differential peaks across treatment conditions with *p*-value < 0.05^62^. ChipPeakAnno^63^ was used to assess overlap of differential peaks and relate peaks to annotated transcription start sites using default options except --PeakLocForDistance = “middle”. NFκB motifs within ATAC-seq peaks were defined by the consensus sequence GGRNNN(N)YCC.

## Supplementary information

Supplemental Figures

Supplementary Table 1

Supplementary Table 2

## Data Availability

Sequencing data is publicly available at NIH SRA, https://www.ncbi.nlm.nih.gov/sra, under experiment numbers SRP145599 (RNA-seq) and SRP145626 (ATAC-seq)
