# Supplementary material for: Sequential conditioning-stimulation reveals distinct gene- and stimulus-specific effects of Type I and II IFN on human macrophage functions
Source: Sci Rep. 2019 Mar 27;9:5288. doi: 10.1038/s41598-019-40503-y (PMC6437173; doi:10.1038/s41598-019-40503-y)
Supplement: Supplementary file 1 — Supplemental Figures [file 41598_2019_40503_MOESM1_ESM.pdf]

SUPPLEMENTAL FIGURES

Sequential conditioning-stimulation reveals distinct gene- and stimulus-specific effects of Type I and II IFN on human macrophage functions

Quen Cheng[1,2], Faraz Behzadi[1,3], Supriya Sen[1], Sho Ohta[1], Roberto Spreafico[1,3], Rosane Teles[4], Robert Modlin[1,4], Alexander Hoffmann[1,3,*]

[1] Department of Microbiology, Immunology, and Molecular Genetics, University of California, Los Angeles, CA 90095

[2] Department of Medicine, Division of Infectious Diseases, David Geffen School of Medicine, University of California, Los Angeles, CA 90095

[3] Institute for Quantitative and Computational Biosciences, University of California, Los Angeles, CA 90095

[4] Division of Dermatology, David Geffen School of Medicine, University of California, Los Angeles, CA 90095

[*] address correspondence to ahoffmann@ucla.edu

a

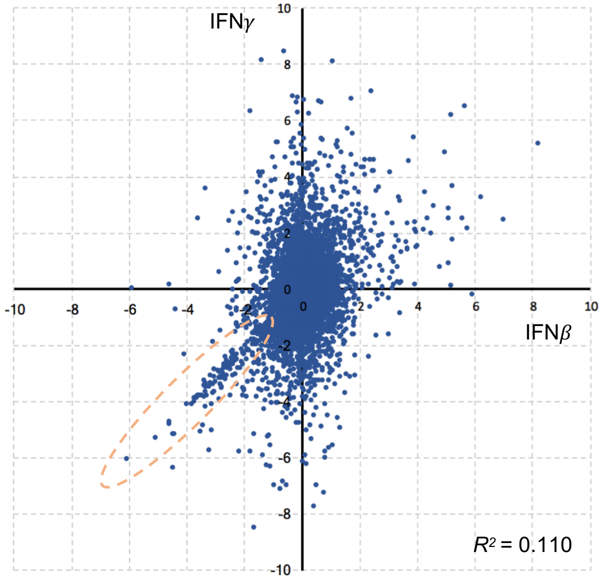

b

GO term	*p*-value
Cell cycle	6.86×10^{-54}
Mitotic cell cycle	4.34×10^{-42}
Chromosome segregation	1.25×10^{-32}
Cell division	3.77×10^{-32}
Chromosome organization	1.67×10^{-25}

Figure S1: IFN effects on basal gene expression **(a)** Scatter plot of IFNβ vs IFN-γ conditioning effect on basal (pre-stimulation) expression, each point representing one gene. Axes are log(2) fold-change of IFN-conditioned gene expression over naïve gene expression. Concordantly down-regulated genes are highlighted. **(b)** Gene ontology analysis of concordantly down-regulated genes, top five non-redundant results.

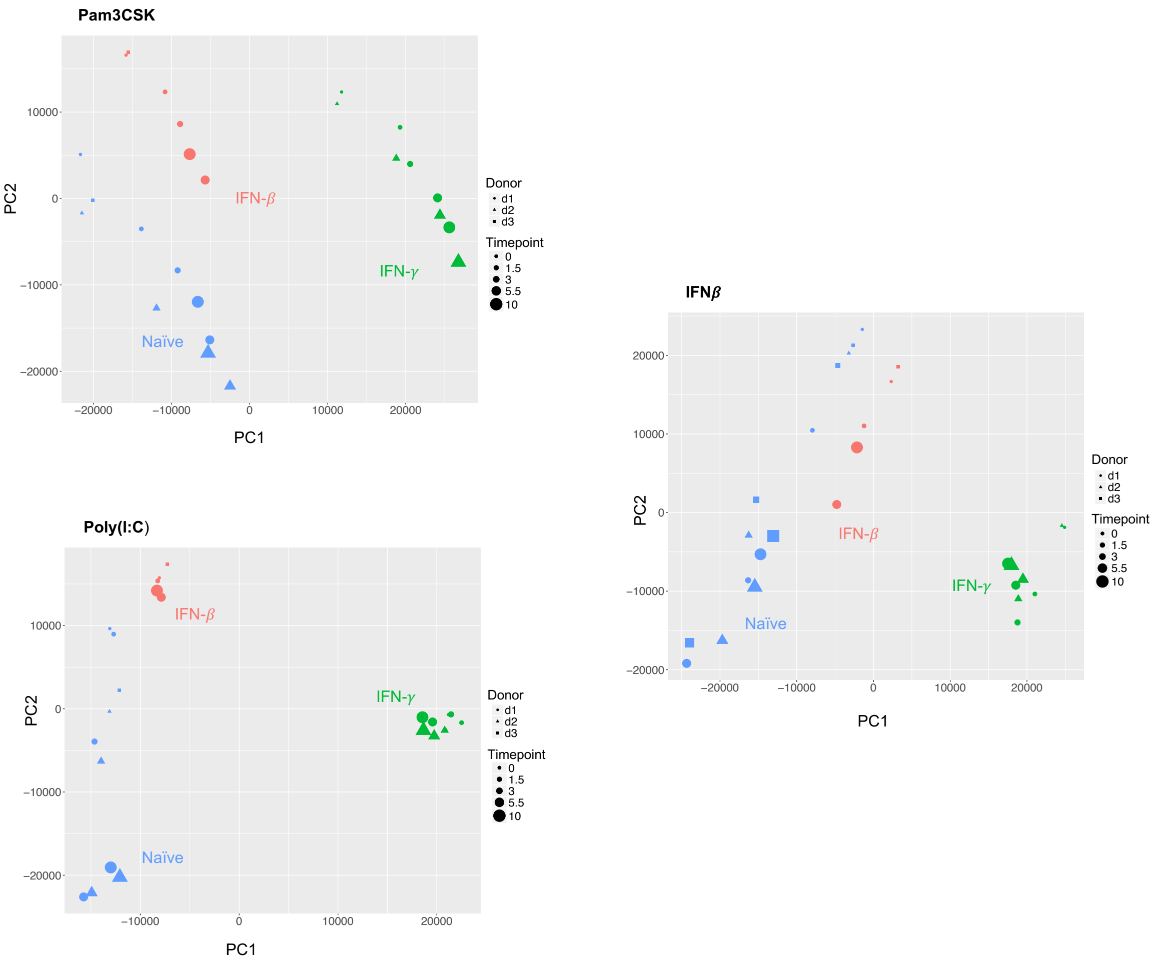

Figure S2: PCA plots for Pam3CSK, poly(I:C), and IFN-β stimulation conditions illustrate the differential effect of IFNβ vs IFNγ conditioning on stimulus-responsive gene expression. Biological replicates are denoted by shape, and time points are denoted by size with the smallest markers showing the effect of IFN conditioning without second stimulation.

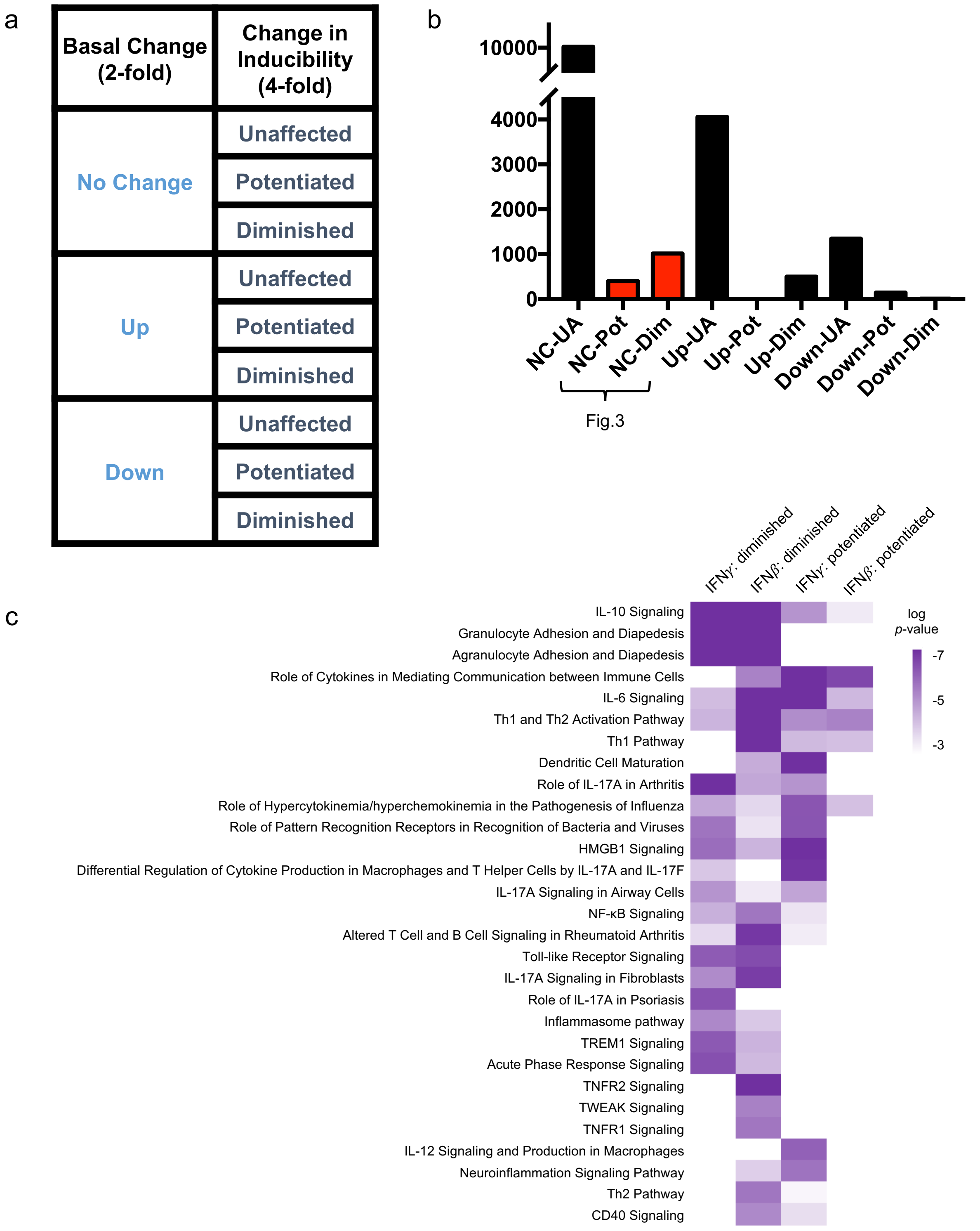

Figure S3: Nine category analysis **(a)** Gene expression thresholds used to categorize every combination of conditioning regimen, stimulus, and gene into nine categories. **(b)** Distribution of the 17,540 cases across the nine categories. Highlighted categories were further analyzed in Fig.3. **(c)** Ingenuity Pathway Analysis of the genes falling into “no change / diminished” and “down / diminished” or “no change / potentiated” and “up / potentiated” categories, separated by IFN conditioning. The canonical pathways with log(*p*-value) < -5 in at least one category are ordered by hierarchical clustering.

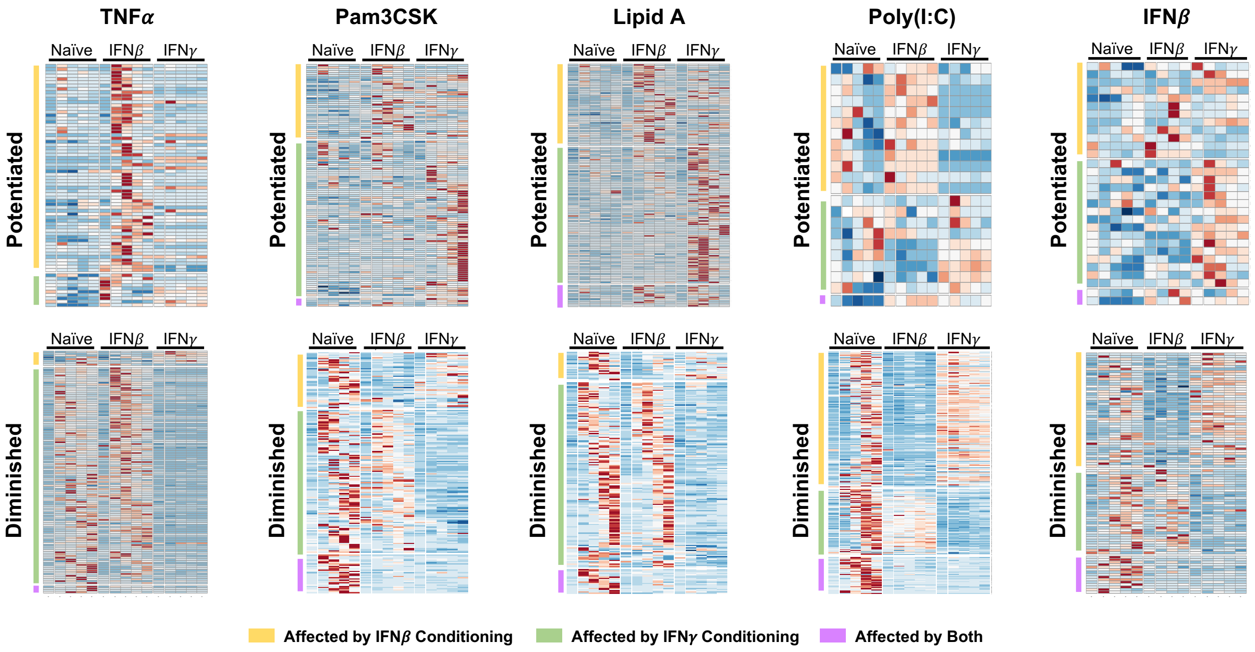

Figure S4: Heatmaps of all genes that are unaffected by IFN alone but have a potentiated (top) or diminished (bottom) response to second stimulation, clustered by whether the criteria are met in IFNβ conditioning, IFNγ conditioning, or both.

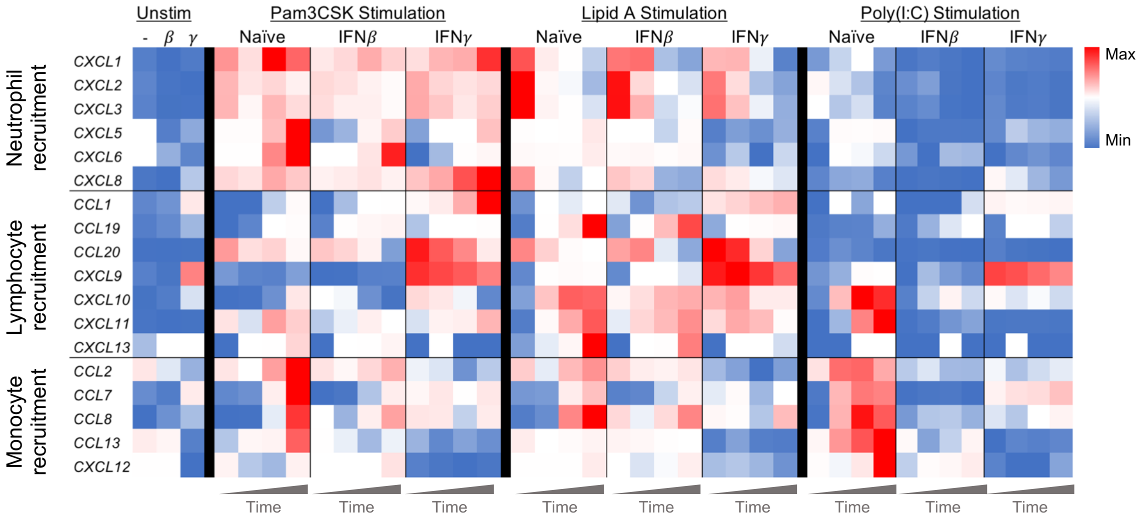

Figure S5: Heat map of stimulus-responsive chemokines, grouped by primary cell type recruited (Mantovani et al., *Nat Rev Immunology* 2006), and the effect of IFN conditioning on their response to TLR ligands Pam3CSK, Lipid A, and poly(I:C).
